# Supplementary material for: Testing the existence of an unadmixed ancestor from a specific population t generations ago
Source: PLoS One. 2022 Aug 12;17(8):e0271097. doi: 10.1371/journal.pone.0271097 (PMC9374268; doi:10.1371/journal.pone.0271097)
Supplement: S1 File — (ZIP) [file pone.0271097.s001.zip › supp.pdf]

Supplementary material

1 Proof of theorem 1.1

Let us consider the hypotheses tests

$$\begin{aligned} &H_0\colon a^0 \text{ has, at least, one } \mathcal{P}_1\text{-complete ancestor, } t \text{ generations ago.}\\ &H_1\colon a^0 \text{ has no } \mathcal{P}_1\text{-complete ancestors, } t \text{ generations ago.} \end{aligned} \tag{1}$$

and

$$\begin{aligned} \overline{H}_0\colon &a^0 \text{ has exactly one } \mathcal{P}_1\text{-complete ancestor, } t \text{ generations ago.}\\ &\text{The other ancestors are } \mathcal{P}_2\text{-complete.}\\ H_1\colon &a^0 \text{ has no } \mathcal{P}_1\text{-complete ancestors, } t \text{ generations ago.} \end{aligned} \tag{2}$$

Then the following theorem holds:

Theorem 1.1. *Let p be any of the four p -values defined for the hypothesis test 2 (p_{mm}, p_{sm}, p_{ms}, p_{ss}). Then, $CR = \{p \leq \alpha\}$ is a critical region for the test 1 with probability $\beta \leq \alpha$.*

Proof. In this section, we will use uppercase to denote random variables, and lowercase to denote values or samples of the random variables. For example, if the ancestors of a^0 were denoted by a^t, we will denote by A^t the random variable that represents how to sample a^t. We will always clarify if the support of A^t is H_0, $\overline{H}_0$ or other space. In the same fashion, if we denoted the chromosome pair statistics by m_i, we will denote by M_i the random variable that represents how to sample m_i.

We introduce now another important source of randomness in this process. Let us fix an individual a_0, and we know that a^t are their ancestors t generations ago. In order to build the family tree, we need to know all the meiosis information from generation t to generation 1, in order to recreate all the pairings from generation t to generation 0. We will denote by r the vector containing all this information, and R the random variable from which r is sampled (this objects also depend on t, but we want to keep the notation as simple as possible). Although r is a complex object, we know the distribution function of R under the meiosis model we are using. It is reasonable to assume that the random variables R and A^t are independent.

As stated before, we would like to know the distribution function of M_i under H_0, for $i = 1, \ldots, 22$. Our strategy is to approximate it using Monte Carlo simulations.

Using that the only sources of randomness in our model are the independent random variables R (recombination sites and starting chromosomes when modeling meiosis) and A^t (genetic information of ancestors t generations ago), we know that there exist a random mapping representation

$$M_i = g_i(R^t, A^t). \tag{3}$$

Where M_i denotes one of the chromosome statistics defined before. Although we can simulate R, we have no information about the distribution function of A^t, meaning that we can not simulate M_i under H_0. However, under $\overline{H}_0$,

$$\overline{M}_i = g_i(R^t, \overline{a}^t). \tag{4}$$

where a_1^t is a $\mathcal{P}_1$-complete ancestor, and a_i^t are $\mathcal{P}_2$-complete ancestors, for $i = 2, \dots, 2^t$. Now that we avoided the problem of A^t, we are able to simulate. The first step is to prove that

$$\overline{M}_i \overset{(s)}{\leq} M_i \tag{5}$$

where $\overset{(s)}{\leq}$ denotes stochastic dominance.

Let c_i^j the jth chromosome of the ith chromosome pair of A^0, where$j = 1, 2$, $i = 1, \dots, 22$. We observe that there exists a partition $X_i^j = \{x_0 = 0, x_1, \dots, x_m, x_{m+1} = L_i\}$ such that each segment $[x_k, x_{k+1}]$ is inherited from one of the ancestors from t generations ago. We will consider the minimum among such partitions (in the sense of number of segments), which only depends on R.

Now, let us consider two individuals a^0, b^0. We will say that $a^0 \leq_1 b^0$ if any $\mathcal{P}_1$-tract $[x_1, x_2] \subset c_i^j$ for a^0 is also a $\mathcal{P}_1$-tract for b^0, for every $i = 1, \dots, 22$, $j = 1, 2$. In general, we say that $a^t \leq_1 b^t$ if $a_k^t \leq_1 b_k^t$ for $k = 1, \dots, 2^t$. We observe that, if $a^t \in H_0$, then $\overline{a} \leq_1 a^t$ (we are using the fact that we can reorder both familiy trees in such a way that a_1^t and $\overline{a}_1^t$ are both $\mathcal{P}_1$-complete).

In this setting, we know that there exists a random mapping representation f such that $A^0 = f(A^t, R)$. Intuitively this means that, if we know A^t and R, we know how to sample an individual a^0 using the random variable A^0.

The proof of this theorem is based on the following lemma:

Lemma 1.1. *Let $t \in \mathbb{N}$, and let r be a sample from R. If $a^t \leq_1 b^t$, then $a^0 = f(a^t, r) \leq_1 f(b^t, r) = b^0$.*

Proof. Let us focus in c_i^j. Let $[w, z] \subset c_i^j$ be a $\mathcal{P}_1$-tract for a^0. Next we consider $[w, z] \cap X_{i,j}$, obtaining a decomposition of $[w, z]$ into $\mathcal{P}_1$-tracts inherited from different ancestors in a^t. We know that r is common for both a^0 and b^0, meaning that $X_{i,j}$ is a common partition; if $[x_i, x_{i+1}]$ was inherited from a_k^t for a^0, then $[x_i, x_{i+1}]$ was inherited from b_k^t for b^0. As $a^t \leq_1 b^t$, then every segment of the decomposition of $[w, z]$ in b^0 is also a $\mathcal{P}_1$-tract, and we conclude that $[w, z]$ is also a $\mathcal{P}_1$-tract for b^0, and thus $a^0 \leq b^0$. □

We conclude that

- If m_i^a is the maximum length among $\mathcal{P}_1$-tracts for a^0 in chromosome i, then the corresponding tract is contained in a $\mathcal{P}_1$-tract for b^0, meaning that $m_i^b \geq m_i^a$.
- Likewise, the sum of lengths of all $\mathcal{P}_1$ tracts in chromosome j for a^0 will be smaller than the sum of lengths of all $\mathcal{P}_1$-tracts in chromosome j for b^0. We conclude that $m_i^b \geq m_i^a$.

□
