# Supplementary material for: Testing the existence of an unadmixed ancestor from a specific population t generations ago
Source: PLoS One. 2022 Aug 12;17(8):e0271097. doi: 10.1371/journal.pone.0271097 (PMC9374268; doi:10.1371/journal.pone.0271097)
Supplement: Attachment — Submitted filename: response_reviewers.pdf [file pone.0271097.s002.pdf]

Response to Reviewers

May 22, 2022

We would like to thank the Editor for taking care of our manuscript *Testing the existence of an unadmixed ancestor from a specific population* **t** *generations ago* (PONE-D-21-38583), and the Reviewers for all their comments and suggestions. We have now produced a new version of the article, taking every comment into consideration, improving several parts of our work as a consequence.

Please, let us know if there is anything else we can do to improve our work.

Kind regards,
Gabriel Illanes.

1 Editor

1. *Please ensure that your manuscript meets PLOS ONE's style requirements, including those for file naming.*

   Done. We think the reviewers' comments also helped in this regard. LaTeX does not compile .tiff files, so we will also include the .png figures in the resubmission, just in case. If anything else needs to be fixed, please let us know and we will do it.

2. *Please update your submission to use the PLOS LaTeX template.*

   Done.

3. *We note that you have provided funding information that is not currently declared in your Funding Statement. However, funding information should not appear in the Acknowledgments section or other areas of your manuscript. We will only publish funding information present in the Funding Statement section of the online submission form.*

   Done.

2 Reviewer 1:

1. *"Here, we have developed a hypothesis test to assess whether it is likely that one of the individual's ancestors t generations ago was an unadmixed ancestor (e.g. complete individuals genome only one ancestry), given a fixed number t of generations and the length of the ancestry-specific tracts for every autosome" How it is different than "Determining the Generation at Which an Individual has a Complete Native Ancestor With High Probability" section in Indigenous Ancestry and Admixture in the Uruguayan Population Spangenberg etal., published in Frontiers in Genetics in September 2021. There is a need to cite Spangenberg etal. at this point. Also please clarify the additional information the current work is adding to the previously published one.*

   The reviewer raises a very good point. This manuscript was intended to be published together with Spangenberg et al manuscript. Due to different circumstances (technical and personal)

it was not possible. Spangenberg's article applied this test to indigenous ancestry genomic data and was able to obtain interesting results. However, the specifics and theory of the test was not published (indeed, only the preprint of this current article was cited). In this current article, the test is presented, developed and explored (this was not done before). The theory is given, and the advantages and also caveats are explained. Additional data sets are also used (genomic data for ten individuals with African ancestry) to assess its usability.

2. *The line According to historical records, most Uruguayan Amerindian were exterminated in 1831 needs reference.*

   Done.

3. *Introduction could be more informative; for example; the authors did not talk about the already available mtDNA and Y-chromosome based analysis on these individuals. They also did not talk about what already had been established by the previous analysis about the Uruguayan population (admixture, TreeMix etc). All these additions will improve the readability of the manuscript as the current journal targets a wide range of readers.*

   The reviewer presented a very good point so the last paragraph of the introduction was properly modified to include previous results.

4. *In heading add 's' to notation*

   Done

5. *We will assume that a_0 and all "her"— I suggest to write as gender neutral as it might lead to confusion that the analysis might have something to discuss only the maternal ancestry.*

   Done.

6. *In Definition 2.4 –For a given $\lambda \in \Lambda$, we say that an individual a is $\mathcal{P}_\lambda$- complete if all "her" chromosomes are $\mathcal{P}_\lambda$-tracts*

   Done. In the same spirit, also changed 'daughter' for 'offspring', and 'mother' for 'parent'.

7. *The hypothesis test 'Our strategy, then, is to focus on a " borderline" ' replace " before borderline with " case of H_0, where we can fix the ancestors' pedigree*

   Done.

8. *The complete essence of the paper relies on this section. So, I strongly recommend a schematic diagram for this section in addition to what authors provided.*

   Done. Added an algorithm summarising the test process at the end of the 'Combining the chromosome scores into a test statistic' subsection.

9. *Please do cite and better write that the mathematical model you are following is in concordance with previously published article.*

   The methodology followed by Spangenberg et. al. was the methodology developed by this work, not the other way around. Indeed, Spangenberg et. al. cites the preprint of this work when following this test's methodology.

10. *Please keep all the figure legends at the end.*

    Done.

11. *Remove an extra "." in legend of Figure 1 "Recombination ...... P2-complete (blue). chromosome."*

    Done.

12. *"As it possess the Markov property, it is easier to develop mathematical models and tests; however, it fails to capture some structures when we work at an individual level, with small values of t." What structures exactly??*

    Done. An example is provided right after that statement.

13. *In all the figures 3 to 9 please label X and Y axes clearly. Also the legends should be self-explanatory.*

    Legends of figures were updated and expanded. However, all axes were already clearly labeled, except Y-axes of histograms, where we find that "frequency" or similar names are underwhelming, so we did not know how to improve upon it. However, we would gladly apply any concrete suggestion the reviewer proposes.

14. *References needs to be checked properly*

    Done.

15. *It would be quite interesting if this model could be tested on some of the other known unadmixed populations and would enhance the strength of the paper.*

    Indeed it would be very interesting to apply the test in other populations. Here, we tested it on 20 individuals, 10 indigenous descendants and 10 African descendants, both groups with enough meta-information to assess the plausibility of results. We know the approximate age of each individual, sex, we have genealogies (family history) and genomic ancestry estimations. We are able to infer whether results make sense or not. If we would like to include another human population, such as 1000G individuals (AMR group) we would lack such meta information that gives us some security on the drawn conclusions.

16. *In figures 6-9, please elaborate observation regarding all the individuals e.g individuals 12,14,15,16 and 19 reject hypothesis*

    Done. We now explain that rejecting one of the four test for a given t and individual is enough to reject the null hypothesis, and provide a better explanation of the empirical results.

17. *Similarly also discuss other individuals especially individual 5*

    Done.

18. *While the codes* `https://github.com/gabriel-illanes/Ancestors_test` *are publicly available; Variants data unable to be retrieved from the* `http://urugenomes.org/lovd/variants` *probably due to some technical. Please ensure the public availability of the data.*

    Variant data was uploaded into that site, but we were forced to make it unavailable due to the new revision of the data protection law in Uruguay. We have discussed the issue with institutional lawyers and apparently we are free to upload the data again.

    In the meantime, the data is available in `https://filebox.cmat.edu.uy/s/wbRwDHxS8E28m3m`, and will be so until the data can be uploaded again to the Urugenomes website.

3 Reviewer 2:

1. *"However, I found it confusing that the empirical analysis is on a group of individuals for whom a reference population is necessarily lacking (because no non-admixed individuals are living), yet a reference population is provided. Some clarity on how the reference panel of data were determined should be provided. Due to this questionable reference comparison, the questionable statistics, and the lack of data availability, I am unsure whether to believe the conclusions in this paper. However, some of this may be solvable with clearer and more comprehensive explanations."*

The reviewer poses a good question. The main idea is that the native-American reference ancestor is not necessarily Charrúa, but a representative of a native-American individual that lived on the region. So, when coloring an individual's chromosome, a Charrúan tract will be closer to the native-American reference ancestor than to the European or African ones, and thus will be correctly assigned to native-American.

However, it is important to note that data-related questions are within the scope of Spangenberg et. al. work, not this work. In this work, the goal is to develop a hypothesis test that can help works such as Spangenberg et. al. (that was the main motivation for this work).

2. *"There is no explanation of the simulations other than a reference to code. The code lacks comments that would explain the analysis. Additionally, the code refers to data not contained in the github repo. There's a link to click for the variants but when I do this it says "No variants found" so it's unclear how I would obtain data to run the code so I could make my own attempt to sort out what the code does (which I should not have to do). It's unclear if these were supposed to be simulated data or human genome variants. Either way, I do not see any data."*

   Done. The github repo was updated with comments for basic usage, the jcode.jl file (with the main functions for running every test) is now commented and has the code for obtaining the empirical data results, and the power results for all three scenarios (which is the focus of this work). Also, the rcode.R outputs were also uploaded to the github repo, so there is no need of the variant data to try the tests code out.

   Regarding the data, variant data was uploaded into that site, but we were forced to make it unavailable due to the new revision of the data protection law in Uruguay. We have discussed the issue with institutional lawyers and apparently we are free to upload the data again.

   In the meantime, the data is available in `https://filebox.cmat.edu.uy/s/wbRwDHxS8E28m3m`, and will be so until the data can be uploaded again to the Urugenomes website.

3. *"There should be further details on variant identification including tools, versions, and parameters."*

   If there is anything that was not uploaded in `https://filebox.cmat.edu.uy/s/wbRwDHxS8E28m3m` that is needed, please let us know.

4. *"Figures 6-9 should not be line graphs. These can be combined into a multi panel figure with a much-expanded caption that provides more detailed explanation."*

   Done. We agree with the reviewer that the lines could be confusing. We thought it was important to keep track of the results for the same t and different individuals. We do this now using different shapes. Legends are now unified and expanded.

5. *"This manuscript has some duplication with Spangenberg et al. BioRxiv 2021 -* `https://www.biorxiv.org/content/10.1101/2021.06.09.447750v1.full` *, which claims to develop the same test described in the manuscript. Assuming that Spangenberg et al. correct their manuscript to state that they only use the test in this manuscript, it is still problematic that both manuscripts describe the empirical results of the test using this particular set of Uruguayan individuals. Given the nature of this manuscript using empirical data from known pedigrees (e.g. for D. melanogaster) would be far more logical."*

   It is important to clarify that Spangenberg et. al. follows this work when presenting the hypothesis test results, and not the other way around. Indeed, Spangenberg et. al. cites this work's preprint when following the test methodology. And we recall that the scopes of this work and Spangenberg et. al. are different. Furthermore, this work also present results for 10 Uruguayan individuals with declared African ancestry, that were not included in Spangenberg et. al.

6. *"In the concluding paragraph where does the biological expectation of " a complete Amerindian ancestor only 2 generations ago " come from?"*

   We agree with the reviewer, the statement is too ambitious considering the test's power, and thus it was removed.

7. *"The paper states " Individuals 12, 14, 15, 16, 19 and 20 reject the hypothesis for the presence of a complete Amerindian ancestor* $t = 5$, $t = 4$ *and* $t = 3$ *generations ago. " but p values near 0 appear for* $t = 2$ *for nearly all individuals (although this depends on the test). This would support* H_1 *that there were no complete ancestors at* $t = 0$. *High p values for other t are shown in the figures. I would have drawn the conclusion that for some individuals you can't reject a complete ancestor at t=3, and for almost all at t=4, and all but 1 at t=5 (at least looking at Fig 6 - since you give four different tests I could use some guidance on which to believe when they conflict)."*

   We agree with the reviewer, the statement is confusing. The results were rewritten and hopefully now they are clearer.
